# Impact of High-Dose Irradiation on Human iPSC-Derived Cardiomyocytes Using Multi-Electrode Arrays: Implications for the Antiarrhythmic Effects of Cardiac Radioablation

**DOI:** 10.3390/ijms23010351

**Published:** 2021-12-29

**Authors:** Jae Sik Kim, Seong Woo Choi, Yun-Gwi Park, Sung Joon Kim, Chang Heon Choi, Myung-Jin Cha, Ji Hyun Chang

**Affiliations:** 1 Department of Radiation Oncology, Seoul National University College of Medicine, Seoul 03080, Korea; icarusky@snu.ac.kr (J.S.K.); dm140@naver.com (C.H.C.); 2 Department of Radiation Oncology, Kyung Hee University Hospital at Gangdong, Seoul 05278, Korea; 3 Department of Physiology, Dongguk University College of Medicine, Gyeongju 38066, Korea; physiolcsw@dongguk.ac.kr; 4 Stem Cell Research Institute, T&R Biofab Co., Ltd., Siheung 15073, Korea; ygpark@tnrbiofab.com; 5 Department of Physiology & Ischemic/Hypoxic Disease Institute, Seoul National University College of Medicine, Seoul 03080, Korea; sjoonkim@snu.ac.kr; 6 Department of Radiation Oncology, Seoul National University Hospital, Seoul 03080, Korea; 7 Division of Cardiology, Department of Internal Medicine, Asan Medical Center, University of Ulsan College of Medicine, Seoul 05505, Korea

**Keywords:** cardiac radioablation, electrophysiological alternation, human induced pluripotent stem cell-derived cardiomyocyte, multielectrode array, radiotherapy

## Abstract

Cardiac radioablation is emerging as an alternative option for refractory ventricular arrhythmias. However, the immediate acute effect of high-dose irradiation on human cardiomyocytes remains poorly known. We measured the electrical activities of human induced pluripotent stem cell-derived cardiomyocytes (iPSC-CMs) upon irradiation with 0, 20, 25, 30, 40, and 50 Gy using a multi-electrode array, and cardiomyocyte function gene levels were evaluated. iPSC-CMs showed to recover their electrophysiological activities (total active electrode, spike amplitude and slope, and corrected field potential duration) within 3–6 h from the acute effects of high-dose irradiation. The beat rate immediately increased until 3 h after irradiation, but it steadily decreased afterward. Conduction velocity slowed in cells irradiated with ≥25 Gy until 6–12 h and recovered within 24 h; notably, 20 and 25 Gy-treated groups showed subsequent continuous increase. At day 7 post-irradiation, except for *cTnT*, cardiomyocyte function gene levels increased with increasing irradiation dose, but uniquely peaked at 25–30 Gy. Altogether, high-dose irradiation immediately and reversibly modifies the electrical conduction of cardiomyocytes. Thus, compensatory mechanisms at the cellular level may be activated after the high-dose irradiation acute effects, thereby, contributing to the immediate antiarrhythmic outcome of cardiac radioablation for refractory ventricular arrhythmias.

## 1. Introduction

Sudden cardiac death accounts for more than 60% of all cardiovascular-related deaths, raising crucial health issues individually but also socioeconomically [1]. Among several risk factors of this global leading death cause, ventricular arrhythmias (VAs) are an important problem [1]. In the past decades, considerable therapeutic advancement has been made for VAs, including antiarrhythmic drugs or catheter ablation that successfully manage these events, and implantable cardioverter-defibrillator that reduces sudden cardiac deaths [2,3]. However, up to about 50% of treated patients experience VA recurrence [4].

Stereotactic body radiotherapy (SBRT) has been in the spotlight as an alternative noninvasive approach for refractory VAs [5]. It is widely used as a radiotherapy procedure to deliver large radiation doses with high precision to target lesions, especially tumors [6]. As a result of the development of noninvasive cardiac imaging to delineate myocardial scars causing VAs [7], SBRT has become a feasible approach to ablate arrhythmia [5]. We previously reported early cardiac in vivo changes induced by radioablation in rats [8]; nonetheless, few studies describing the molecular and cellular mechanisms of these acute effects are available. Moreover, given the high radiation dose being administered, the safety of adjacent normal cardiomyocytes should be investigated and warranted.

In this study, we used human induced pluripotent stem cell-derived cardiomyocytes (iPSC-CMs) and a multielectrode array (MEA) recording system to study temporal electrophysiological alternations induced by radioablation at the cellular level without any influence of the extracellular environment. Human iPSC-CMs offer an unprecedented opportunity to investigate the complicated electrophysiological activities of human cardiomyocytes, as they express all major ion channels of human cardiomyocytes [9]. The MEA recording system captures noninvasively extracellular field potentials, correlating to action potentials, across the human iPSC-CM monolayers simultaneously with real-time high throughput [10]. Using this methodology, field potential duration (FPD), considered as the QT interval in the electrocardiogram, can be analyzed and its prolongation after intervention can provide information on the potential risk for cardiotoxicities [11]. Currently, human iPSC-CMs in combination with MEA have emerged as a relevant in vitro model of cardiotoxicity screening [12].

In the present study, we assessed seven electrophysiological parameters, including the number of the total active electrodes, spike amplitude and slope, FPD, corrected FPD (FPDc), beat rate, and conduction velocity (CV), until 7 days after high-dose irradiation. We witnessed dynamic changes in these features during the study period, which were altered immediately in a few hours, but some of them finally recovered to the baselines. FPD was prolonged in all irradiated groups except for 50 Gy, and the beat rate decreased after immediate increase following high-dose irradiation. CV was increased subsequently after an immediate decrease in 20 and 25 Gy groups. Changes in total active electrode, spike amplitude, and spike slope were dependent on irradiation dose. Moreover, high-dose irradiation led to increased expression of genes relating to cardiomyocyte functions.

## 2. Results

### 2.1. Immediate Electrophysiological Effects of High-Dose Irradiation on Human iPSC-CMs

**Figure 1:**
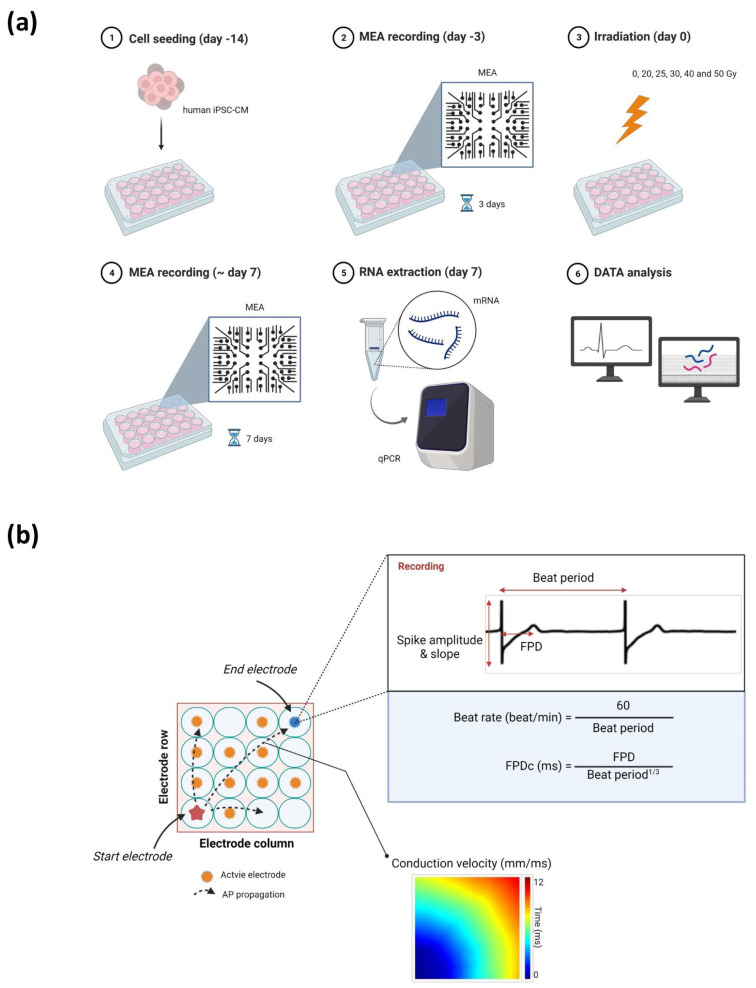
Illustration of the (**a**) experimental protocol and (**b**) parameters assessed using a multielectrode array. Abbreviations: AP, action potential; FPD, field potential duration; FPDc, corrected field potential duration; iPSC-CM, induced pluripotent stem cell-derived cardiomyocyte; MEA, multielectrode array; qPCR, quantitative polymerase chain reaction.

**Figure 2:**
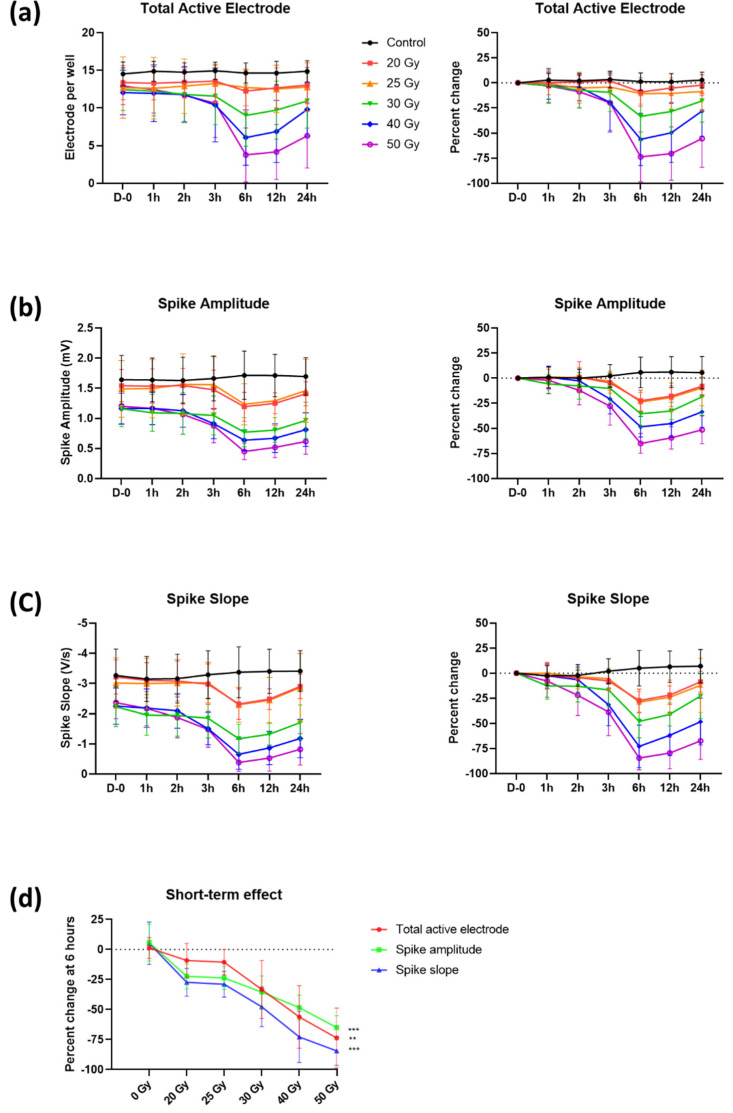
Temporal changes of (**a**) total active electrode, (**b**) spike amplitude, and (**c**) spike slope with percent changes within 24 h after high-dose irradiation. (**d**) Dose–response relationship of these three parameters at 6 h. *p*-values of dose–response relationship were determined by Pearson’s correlation coefficient; ** *p* < 0.010 and *** *p* < 0.001. Values are depicted as the mean ± standard deviation of *n*_wells_ = 20–24.

**Figure 3:**
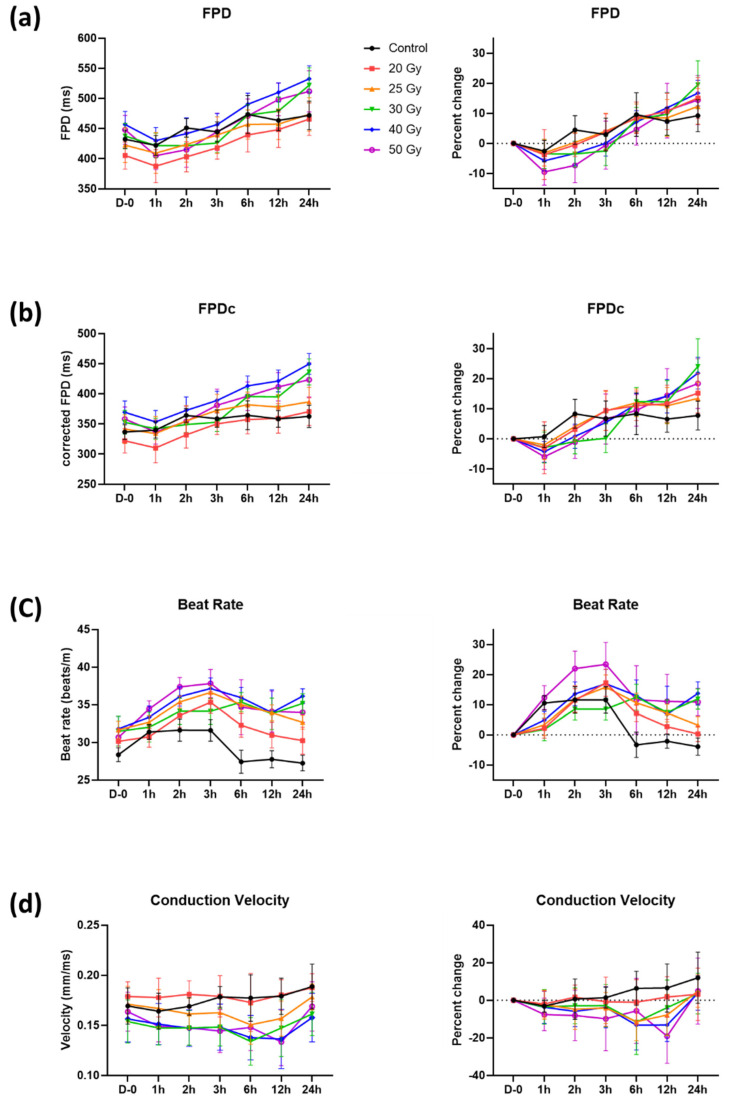
Temporal changes of (**a**) field potential duration (FPD), (**b**) corrected FPD (FPDc), (**c**) beat rate, and (**d**) conduction velocity with percent changes within 24 h after high-dose irradiation. Values are depicted as the mean ± standard deviation of *n*_wells_ = 20–24.

We first assessed the electrical activities of human iPSC-CMs using the MEA recording system for 7 days after irradiation with 0, 20, 25, 30, 40, and 50 Gy (Figure 1a). We analyzed the electrophysiological alterations within 24 h as an acute response of cardiomyocyte functions upon high-dose irradiation (Figure 2 and Figure 3). We evaluated percent changes as the outcomes of the records, defined by differences from the baseline (day 0) of each radiation dose group as all the baseline parameters on day 0 were relatively dispersed among different radiation dose groups.

Total active electrodes (>300 µV for spike threshold) per well, spike amplitude, and spike slope (Figure 1b) began to decrease 2 h after irradiation (Figure 2a–c). These declines were observed until 6 h (in the group of 50 Gy: total active electrodes, −73.7 ± 24.9%; spike amplitude, −65.0 ± 9.9%; and spike slope, −84.4 ± 12.0%) and then slightly increased. The reduction of the spike amplitude and slope led to a decrease in the detected electrodes (i.e., total active electrodes). The changes in these parameters were highly associated with the radiation dose applied. To determine the association between these three parameters and the radiation dose, values of 6 h with the largest percent changes were used (Figure 2d). Total active electrodes, spike amplitude, and spike slope decreased from −9.1 ± 14.1% to −73.7 ± 24.9% (Pearson’s correlation coefficient, *p* < 0.010), from −22.4 ± 10.5% to −65.0 ± 9.9% (*p* < 0.001), and from −27.4 ± 11.6% to −84.4 ± 12.0% (*p* < 0.001), respectively, as radiation dose increased from 20 to 50 Gy.

Parameters reflecting the duration of action potential repolarization, such as FPD(c) (FPD and FPDc) and beat rates, were assessed (Figure 1b). FPD(c) and beat rate increased by approximately 10%, even in the control group, showing the vulnerability of these parameters. Nonetheless, FPD(c) showed a tendency to slightly decrease at first, especially in the 40 and 50 Gy groups, but eventually increased with time (Figure 3a,b). FPD(c) changes were not dependent on radiation dose. The beat rate increased within 1 h in all groups, including the control group (Figure 3c). This increase returned to the baseline level again in the control, 20, and 25 Gy groups, which were relatively small doses, but remained high in the group of 30 Gy or more.

CV of monolayer cardiomyocytes in each well was analyzed as the distance of action potential propagation per millisecond between the MEA electrodes (Figure 1b). It was decreased between 6 and 12 h in the >20 Gy groups, and the largest reduction was measured at 12 h in the 50 Gy group. However, the reductions were temporary, and the values were restored to basal levels in 24 h.

### 2.2. Recovery of Human iPSC-CMs from Immediate Electrophysiological Alterations Induced by High-Dose Irradiation

**Figure 4:**
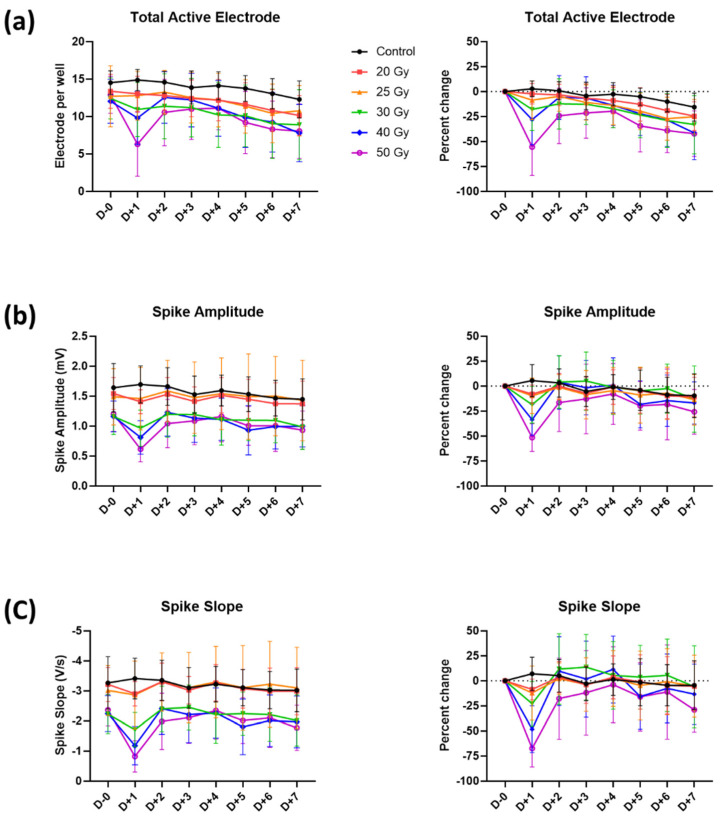
Temporal changes in (**a**) total active electrode, (**b**) spike amplitude, and (**c**) spike slope with percent changes until 7 days after high-dose irradiation. Values are depicted as the mean ± standard deviation of *n*_wells_ = 20–24.

Recovery of the total active electrodes, spike amplitude, and spike slopes from the observed irradiation-induced reductions started after 6 h and continued, reaching almost total recovery by day 2 (Figure 4). For the 50 Gy group, which showed the largest decreases on day 1, the total active electrodes, spike amplitude, and spike slopes increased from −55.5 ± 28.5% to −24.2 ± 27.8%, from −51.4 ± 13.9% to −16.4 ± 29.0%, and from −67.3 ± 18.6% to −17.7 ± 40.5%, respectively. However, total active electrodes on day 7 did not reach the previous baseline values in relatively high dose groups, such as 40 and 50 Gy, in general showing a decreasing trend in all groups (Figure 4a). On day 7, percent changes of 0, 20, 25, 30, 40, and 50 Gy groups were −15.5 ± 14.0%, −24.6 ± 14.4%, −25.1 ± 17.3%, −33.1 ± 29.1%, −41.4 ± 26.7%, and −42.1 ± 22.8%, respectively.

**Figure 5:**
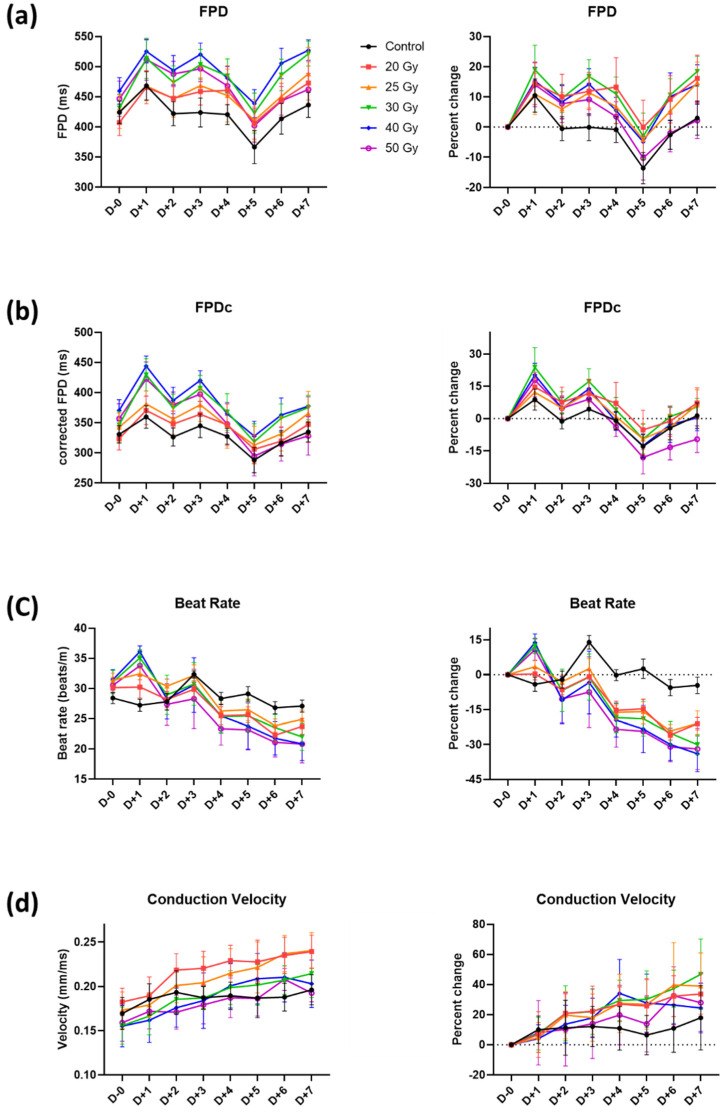
Temporal changes in (**a**) field potential duration (FPD), (**b**) corrected FPD (FPDc), (**c**) beat rate, and (**d**) conduction velocity with percent changes until 7 days after high-dose irradiation. Values are depicted as the mean ± standard deviation of *n*_wells_ = 20–24.

**Figure 6:**
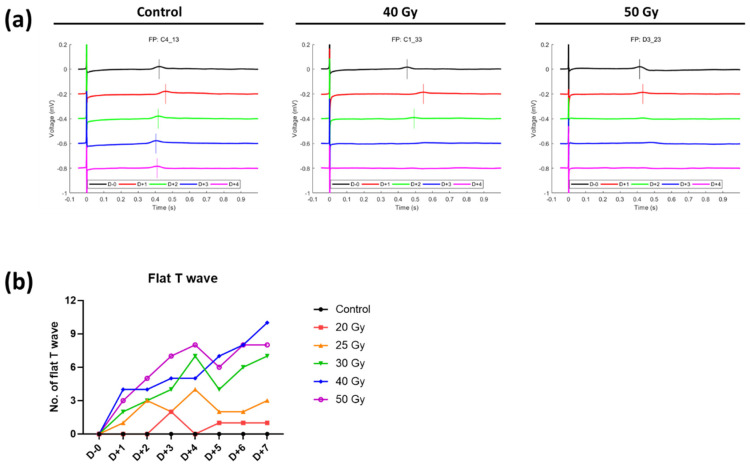
(**a**) Representative beating patterns of cardiomyocytes after high-dose irradiation. (**b**) Number of flat T waves in high-dose irradiated groups (*n*_wells_ = 20–24).

Although fluctuations of FPD(c) in the control group were high, it was found that there was no significant change in FPDc during the 7 days as the values of irradiated groups were changing in a similar pattern to those of the control group (Figure 5a,b). FPDc of the 50 Gy group on day 7 appeared to be shortened compared with that of the control group (percent change on day 7 compared with day 0, −9.6 ± 6.2% and 1.4 ± 5.9% in 50 Gy and control, respectively). Interestingly, the number of T wave flattening tended to increase with time as the irradiation dose increased (Figure 6). Concerning the beat rates of irradiated human iPSC-CMs, they steadily decreased in a dose-independent manner after day 1 and were significantly different from that of the control after day 3 (Figure 5c). Beat rates of 20–50 Gy groups on day 7 were split into two groups: approximately −20% in <30 Gy groups vs. −30% in ≥30 Gy groups.

**Figure 7:**
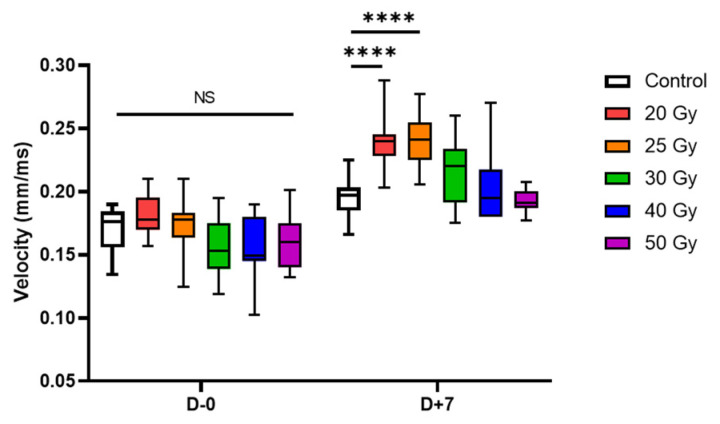
Box-and-whisker plots of conduction velocity on days 0 and 7 after high-dose irradiation (*n*_wells_ = 20–24). Adjusted *p*-values were determined by one-way analysis of variance followed by Dunnett’s multiple comparison test compared with the control group: **** *p* < 0.0001. Abbreviation: NS, not significant.

The restored CV showed a steady increase (Figure 5d). In percent changes, no significant difference compared with control was observed, but concerning absolute values, CV speeded up in the relatively low dose group (20 and 25 Gy). Additionally, although there were no differences in CV between groups on day 0, CV of 20 and 25 Gy groups on day 7 significantly increased compared with that of the control group (*p* < 0.001, Figure 7).

### 2.3. High-Dose Irradiation Promotes Increased Expression of Human iPSC-CM Genes

**Figure 8:**
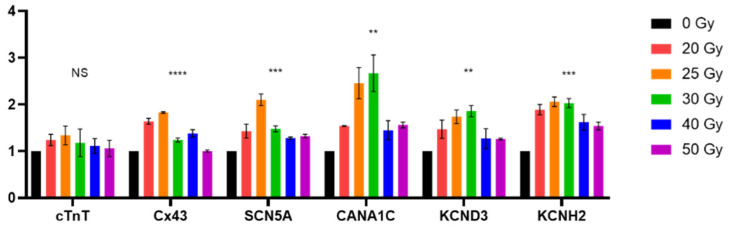
Gene expression level of selective human induced pluripotent stem cell-derived cardiomyocytic marker and ion channel genes 7 days after high-dose irradiation. Values are depicted as the mean ± standard deviation of *n* = 2. All *p*-values were determined by one-way analysis of variance; ** *p* < 0.010, *** *p* < 0.001, and **** *p* < 0.0001. Abbreviation: NS, not significant.

Lastly, we investigated the changes at the molecular level by quantifying mRNA levels of several human iPSC-CM genes 7 days after irradiation (Figure 1a). Cardiomyocyte marker gene (c*TnT*) and major cardiac ion channel genes (*Cx43*, *SCN5A, CANA1C, KCND3*, and *KCNH2*) were selected for this analysis (Figure 8). The expression of these genes, except *cTnT*, tended to increase in the irradiated groups. mRNA levels of lower dose (≤12 Gy) can be found in Figure S1.

## 3. Discussion

SBRT is an emerging therapeutic option for patients with refractory VA [5]. Although the mechanism of suppressing VA is not fully elucidated, recent researches have suggested possible mechanisms [13,14,15]. Radiotherapy-induced fibrotic remodeling and increased CV are presumed to prevent re-entry of cardiac impulses [13,14,15]. However, conducted studies have observed changes over days to months [13,14,15]. Moreover, it takes several months for fibrosis to occur, which is not enough to explain the immediate antiarrhythmic effect of radioablation. In a previous investigation using a rat model, cellular necrosis or apoptosis was not observed after irradiation, but instead, intra- and extracellular edema, dilated capillary, and diffuse mitochondrial damage were noticeable [8]. Nonetheless, these in vivo investigations were mainly focused on the overall changes, including on the surrounding environment, rather than the effect of radiation on the cardiomyocytes themselves. Therefore, in the present study, we aimed to investigate the radiation effect on the heart at the cell level using human iPSC-CMs without the effects of the extracellular environment, such as vascular structures and inflammatory cells.

Our study provided new insights on the electrophysiological changes that occur on human iPSC-CMs over hours to days in response to multiple radiation doses. To the best of our knowledge, this is the first study to describe the direct acute effects of high-dose radiation at the cellular and molecular levels. The main findings were as follows: (1) no acute cell death occurs after high-dose irradiation; (2) immediate electrophysiological changes (decreased excitability, increased FPD, increased beat rate, and cell-to-cell conduction slowing) are restored 1–2 days after irradiation, and the compensatory changes persist until several days; and that (3) these electrophysiological changes may be associated with ion channel function.

In this study, a significant reduction in cardiomyocyte excitability, such as the amplitude and slope of the field potential spike, was observed around 6 h after irradiation with a dose-dependent response (Figure 2). Surprisingly, the reduced excitability, especially in 30–50 Gy-irradiated groups, was almost restored within 2 days (Figure 4). The excitability in human iPSC-CMs requires the activity of the voltage-dependent cardiac sodium channel (Na_V_1.5). Loss-of-function of Nav1.5 occurs frequently in heart disease and causes potentially lethal inheritable arrhythmia syndromes [16,17,18]. Loss of excitability due to Na_V_1.5 defect also causes conduction block diseases [19,20]. Thus, our results suggest that high-dose irradiation may represent a risk for acute arrhythmogenic effects by suppressing the excitability of cardiomyocytes; however, such an event can be subsequently recovered.

FPDc is a beat rate-adjusted FPD (defined as the interval from sodium spike to repolarizing peak), a commonly used parameter for cardiotoxicity screening [11]. As the definition suggests, FPDc is affected by both the FPD and beat rate. Typically, potassium channel blockers prolong the FPDc, whereas calcium channel blockers shorten the FPDc [21]. In our recording, FPD and beat rate were increased so that FPDc was prolonged as an acute response after irradiation, irrespective of the dose applied (Figure 3a–c). Thus, we can expect that potassium channels on cardiomyocytes became faulty after irradiation.

The possible underlying mechanism resulting in loss-of-function of the above sodium channel and potassium channels may be related to the production of reactive oxygen species (ROS) upon irradiation. We did not measure ROS formation in the present study; however, it is well known that ROS is accumulated in cells after high-dose irradiation [22,23,24]. Furthermore, the recovery pattern after radiation-induced rapid change herein observed is identical to that of mitochondrial ROS production and recovery [23]. Mitochondrial ROS is related to Na_V_1.5 channel function suppression [25]. In addition, ROS-induced endoplasmic reticulum stress activates the unfolded protein response, where downstream signaling cascades eventually reduce the activity and expression of cardiac ion channels, leading to arrhythmias [26]. Taken together, it is reasonable to speculate that channel activity may be inhibited by high-dose irradiation through ROS production within hours. The fact that ROS is generated immediately after radiotherapy and is restored after several days can further support both the acute response and recovery pattern herein reported [27]. Further, the persistent compensatory effect after the initial acute, detrimental change may contribute to the antiarrhythmic effect of cardiac radioablation [25,28,29].

The CV between adjacent cells was slightly decreased on the day of irradiation, but it steadily increased until day 7. The CV recovery slope was steeper in the 20–30 Gy groups and slower in the 40–50 Gy groups. This difference can also be explained by the sequela of ROS production, similar to the ion channel functional modulation mentioned above. As mitochondrial oxidative stress is known to decrease CV in some studies [30], the pattern of CV change can also be explained by mitochondrial ROS formation. Additionally, cardiac conduction through cardiomyocytes is regulated by cardiac conduction proteins, specifically connexin43 (Cx43) and Na_V_1.5 in ventricular conduction [19,31,32]. Recently, the mechanism of cardiac radiotherapy was suggested as electrical conduction reprogramming. Ionizing radiation to the heart can increase the levels of cardiac conduction proteins, such as Cx43 and Na_V_1.5, and enhance ventricular conduction [15]. Other previous studies reported lateralization and elevation of Cx43 expression after carbon radiation to the heart [33,34,35]. In the present study, we also evaluated the mRNA levels of *Cx43* and *SCN5A* at day 7 post irradiation (Figure 8). As an alternative to causing structural fibrosis and producing ablative scar on arrhythmogenic tissue, a functional conduction block by CV alternance may be the basic biologic mechanism behind cardiac radioablation.

Noteworthily, we found that the expression of several conduction-related genes was elevated in 20 to 25 or 30 Gy-treated cells, which mimic the conditions used for cardiac radioablation in the clinical setting, and relatively decreased in the higher dose-treated groups. Although we did not identify the exact mechanism of these bell-shaped responses, it is possible that ROS are also involved in this process. In our explanation of the acute response of high-dose irradiation, ROS serve as key molecules to alter the electrophysiological activities of cardiomyocytes. However, they also serve as signaling molecules to compensate for cellular stress [36]. For example, ROS directly trigger transcription factors in prokaryotes for them to adapt to the oxidative environment [37]. In contrast, higher levels of ROS destroy proteins and DNA [38]. Similarly, in our experiment, the expression of genes acting on cardiomyocyte function may have been triggered by ROS until the dose of 30 Gy, whereas higher radiation dosage (over 30 Gy) generated higher levels of ROS, damaging proteins and DNA, thereby, reducing gene expression. This hypothesis may have some limitations, including the inconsistent *cTnT* expression. Nonetheless, these findings are hypothesis-generating and worthy of further investigation.

Regarding the radiation dose used for cardiac radioablation, most clinical studies used 25 Gy administrated in a single fraction and some have also tried lower doses (15–20 Gy) [39,40]. As the optimal dose has not been established, systematic reviews were performed and efforts have been made to build an expert consensus for future practice [41,42].

From a clinical point of view, our findings and explanation focus on cardiac radioablation effect and, thus, may be quite different from classic radiation-induced heart toxicity after radiotherapy concerning radiation dose and timing. High-dose irradiation to the heart during thoracic radiotherapy for lymphoma, breast, lung, or esophageal cancer is known to be related to late cardiac toxicity [43,44]; however, the cardiac toxicity could be observed months or years after irradiation. Moreover, the dose used in conventional radiotherapy is usually fractionated with ≤2 Gy per fraction, as 60 Gy in 30 fractions. This dose is similar to the 16 Gy in a single fraction in simple biological effectiveness calculation (α/β of 3), which is much lower than our experimental doses. Therefore, our result is not directly applicable to clinical radiotherapy for cancer treatment.

Our study has some limitations that should be considered when interpreting its results. We specifically evaluated the effect of high-dose irradiation on cardiomyocytes themselves; thus, we excluded the influence of the extracellular environment. However, given the two-way interaction between them, more intricate outcomes can be expected. In this study, we recorded electrophysiological alternations on cardiomyocytes up to 7 days after high-dose irradiation; however, we cannot guarantee that our findings are sustained during long-term observations. Finally, in our experiment setting, we used healthy cardiomyocytes; thus, further investigations using VA model cells are still required.

In conclusion, the present study was designed to observe the electrophysiological response of human iPSC-CMs from high-dose radiation in the absence of accompanying changes in the extracellular environment. Overall, the collected data successfully revealed the changes in the cardiomyocytes themselves, suggesting that the electrical properties of cardiomyocytes can be remodeled after irradiation without cell death; however, the clear mechanism of electrical conduction reprogramming is yet to be discovered. In conclusion, we demonstrated that the response of a single cell, which could not be discovered in the previous in vivo investigations, clearly plays a role in early arrhythmic change.

## 4. Materials and Methods

### 4.1. Preparation of Human iPSC-CMs

Human iPSC-CMs (Cardiosight-S; NEXEL, Seoul, Korea) were used for MEA and real-time quantitative polymerase chain reaction (qPCR). The cells were prepared according to the manufacturer’s instruction. Briefly, 3 × 10^4^ cells were seeded onto each well coated with fibronectin in the CytoView MEA plate (Axion BioSystems, Atlanta, GA, USA). After 1 h of incubation, Cardiosight-S maintenance medium was added, which was replaced every 2 days. Spontaneous beating was observed within 3 days of incubation at 37 °C and 5% CO_2_. Irradiation was performed 2 weeks after seeding.

### 4.2. Irradiation

Using the Brilliance Big Bore computed tomography simulator (Philips Medical Systems, Cleveland, OH, USA), MEA plates with human iPSC-CMs were scanned for precise calculation of irradiation dose by the Eclipse treatment planning system (Varian Medical Systems, Palo Alto, CA, USA). For irradiation, TrueBeam linac (Varian Medical Systems) was used with a posterior-anterior beam (dose rate 600 MU/min). During all procedures, the plates were placed in a temperature-controlled environment (35–37 °C).

### 4.3. MEA Recording Analysis

Immediately after the irradiation, the plates were placed in the incubator, and MEA recordings were performed with the Maestro Multiwell MEA System (Maestro edge; Axion BioSystems) from 1 h after irradiation. The human iPSC-CM activities, including field potential spike, propagation of excitability, and spontaneous beating, were recorded for 5 min for each plate using AxIS Navigator 2.0.4 (Axion BioSystems). MEA data were analyzed with the AxIS Metric Plotting Tool 2.2.5 and the Cardiac Analysis Tool 2.2.7 (Axion BioSystems). During MEA recording, the absence of cell death after irradiation was confirmed by microscopic examination on each day.

### 4.4. Real-Time qPCR

Total RNA was extracted by adding 1 mL of TRIzol reagent (Thermo Fisher Scientific, Waltham, MA, USA) to cells on a plate, as described in the manufacturer’s instructions. The RNA (1 μg) was subjected to cDNA synthesis with the High-Capacity cDNA Reverse Transcription Kit (Applied Biosystems, Waltham, MA, USA). LightCycler 96 DNA Green Value (Roche, Basel, Switzerland) was used to detect the accumulation of PCR product during cycling with the LightCycler 96 system (Roche). Real-time qPCR was performed in triplicates in at least three independent experiments. Oligonucleotide primers were designed using Primer 3, and their sequences are provided in Table S1. Fold differences in the expression of each gene were calculated for each treatment group using CT values normalized to transcript levels of the housekeeping gene, *GAPDH*, according to the manufacturer’s instructions.

### 4.5. Statistical Analysis

All data were analyzed using Prism 8.3.0 (GraphPad Software, San Diego, CA, USA). Dose–response relationships of the total active electrode and spike amplitude and slope were evaluated by Pearson’s correlation coefficient. One-way analysis of variance was used for comparing CV and cardiomyocyte gene levels. Additionally, Dunnett’s post hoc test was performed to assess differences in CV between the control and irradiated groups. Unless otherwise stated, data are shown as the mean ± standard deviation. A *p*-value less than 0.050 was considered statistically significant. Illustrations in Figure 1 were designed using BioRender (https://app.biorender.com/, accessed date: 23 November 2021).

## Data Availability

The data presented in this study are available on request from the corresponding author.

## Supplementary Materials

The following supporting information can be downloaded at: https://www.mdpi.com/article/10.3390/ijms23010351/s1.
