# Supplementary material for: Impact of High-Dose Irradiation on Human iPSC-Derived Cardiomyocytes Using Multi-Electrode Arrays: Implications for the Antiarrhythmic Effects of Cardiac Radioablation
Source: Int J Mol Sci. 2021 Dec 29;23(1):351. doi: 10.3390/ijms23010351 (PMC8745341; doi:10.3390/ijms23010351)
Supplement: Supplementary file 1 [file ijms-23-00351-s001.zip › ijms-1508238-SI.pdf]

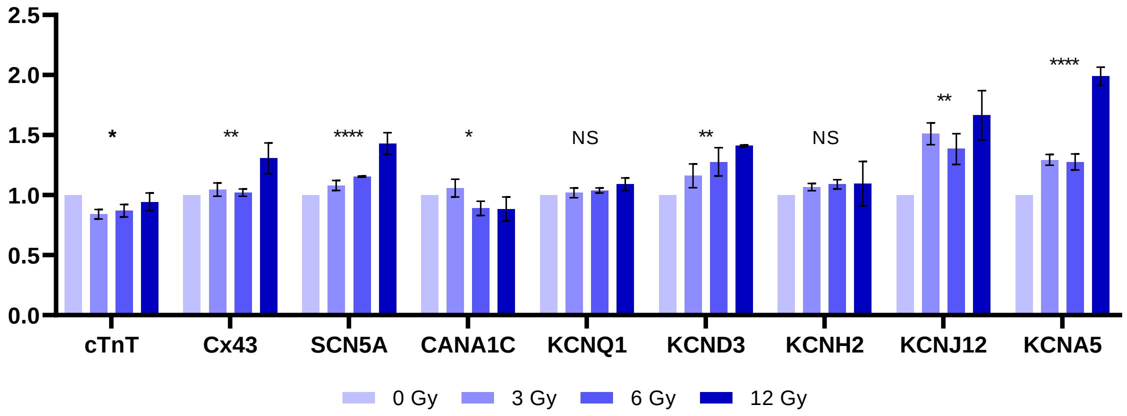

Figure S1. Gene expression of human induced pluripotent stem cell-derived cardiomyocyte marker genes and ion channel genes 7 days after irradiation with 0, 3, 6, and 12 Gy. Values are depicted as mean ± standard deviation of n = 3. All p-values are determined by one-way analysis of variance; *p < 0.050, **p < 0.010, and ****p < 0.0001.

Abbreviation: NS, not significant.

Table S1. Primers used for real-time reverse transcription-polymerase chain reaction

Gene	Forward primer (5′ to 3′)	Reverse primer (5′ to 3′)	Size (bp)	Gene bank No.
**GAPDH**	GTATGACAACAGCCTCAAGA	GTAGAGGCAGGGATGATGT	216	NM_002046
**cTnT**	GGGTTACATCCAGAAGACAG	GTTATAGATGCTCTGCCACA	163	NM_000364
**Cx43**	TGTGGCTGTCAGTACTTTTC	AGTGTGGGTACAGACACAAA	199	AF151980.2
**SCN5A**	TCACCACCTACATCATCATC	GACAGGACCGAATACTCAAT	190	AY038064
**CACNA1C**	CCATCTACAACTACCGTGTG	ACCACGTACCACACTTTGTA	247	NM_1994603
**KCND3**	CTACTACATCGGTCTGGTCA	TGAGGGAGAAGAGAAGAAAG	179	NM_004980
**KCNH2**	CCTCCATCAAGGACAAGTAT	GAAGATGCTAGCGTACATGA	155	AF363636
